# 3,5,6,7,8,3′,4′-Heptamethoxyflavone, a Citrus Flavonoid, Ameliorates Corticosterone-Induced Depression-like Behavior and Restores Brain-Derived Neurotrophic Factor Expression, Neurogenesis, and Neuroplasticity in the Hippocampus

**DOI:** 10.3390/molecules21040541

**Published:** 2016-04-23

**Authors:** Atsushi Sawamoto, Satoshi Okuyama, Kana Yamamoto, Yoshiaki Amakura, Morio Yoshimura, Mitsunari Nakajima, Yoshiko Furukawa

**Affiliations:** 1 Department of Pharmaceutical Pharmacology, College of Pharmaceutical Sciences, Matsuyama University, 4-2 Bunkyo-cho, Matsuyama, Ehime 790-8578, Japan; 46140018@cc.matsuyama-u.ac.jp (A.S.); 66150087@cc.matsuyama-u.ac.jp (K.Y.) mnakajim@cc.matsuyama-u.ac.jp (M.N.); furukawa@cc.matsuyama-u.ac.jp (Y.F.); 2 Department of Pharmacognosy, College of Pharmaceutical Sciences, Matsuyama University, 4-2 Bunkyo-cho, Matsuyama, Ehime 790-8578, Japan; amakura@cc.matsuyama-u.ac.jp (Y.A.); myoshimu@cc.matsuyama-u.ac.jp (M.Y.)

**Keywords:** heptamethoxyflavone, depression, corticosterone, hippocampus, brain-derived neurotrophic factor, neurogenesis

## Abstract

We previously reported that the citrus flavonoid 3,5,6,7,8,3′,4′-heptamethoxyflavone (HMF) increased the expression of brain-derived neurotrophic factor (BDNF) in the hippocampus of a transient global ischemia mouse model. Since the BDNF hypothesis of depression postulates that a reduction in BDNF is directly involved in the pathophysiology of depression, we evaluated the anti-depressive effects of HMF in mice with subcutaneously administered corticosterone at a dose of 20 mg/kg/day for 25 days. We demonstrated that the HMF treatment ameliorated (1) corticosterone-induced body weight loss, (2) corticosterone-induced depression-like behavior, and (3) corticosterone-induced reductions in BDNF production in the hippocampus. We also showed that the HMF treatment restored (4) corticosterone-induced reductions in neurogenesis in the dentate gyrus subgranular zone and (5) corticosterone-induced reductions in the expression levels of phosphorylated calcium-calmodulin-dependent protein kinase II and extracellular signal-regulated kinase1/2. These results suggest that HMF exerts its effects as an anti-depressant drug by inducing the expression of BDNF.

## 1. Introduction

The incidence of depression is increasing in every generation worldwide. A large number of studies have identified genetic factors, environmental factors, and stress as major risk factors for depression [1]. Stress induces the activation of the hypothalamic-pituitary-adrenal (HPA) axis [2], resulting in the over-secretion of glucocorticoids, which, in turn, leads to the depressive symptomatology [3]. Recent studies also showed that decreases in the levels of hippocampal brain-derived neurotrophic factor (BDNF), the most important neurotrophic factor in the brain, correlated with stress-induced depressive behavior; however, treatments with anti-depressants have been suggested to restore BDNF levels [4,5,6]. The BDNF hypothesis of depression was recently proposed based on these findings [7].

**Figure 1:**
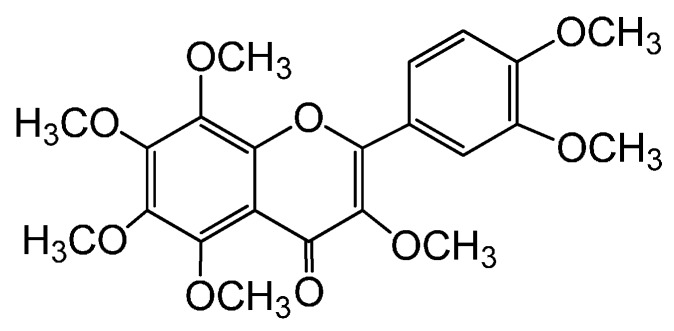
Structure of 3,5,6,7,8,3′,4′-heptamethoxyflavone (HMF).

In addition to increasing evidence to show that BDNF is one of the representative molecules for depression, we previously demonstrated that 3,5,6,7,8,3′,4′-heptamethoxyflavone (HMF; Figure 1), a citrus polymethoxyflavone, has the potential to accelerate the synthesis of BDNF in the hippocampus following ischemia [8,9]. Therefore, we herein determined whether HMF ameliorates depressive-like behavior and depressive disorders in a depression mouse model. In the present study, a depression mice model was developed through the chronic administration of glucocorticoids, which was based on clinical observations that glucocorticoid levels are elevated in depressed patients [3]. Patients with elevated glucocorticoid levels (for example, by the administration of a high dose of corticosteroids) exhibit a depression-like state [10]. We previously reported that the repeated administration of corticosterone at a dose of 20 mg/kg for 3 weeks induced depressive conditions in mice [11]. These mice exhibited 1) depression-like behavior, 2) reduced body weight, and 3) decreases in the phosphorylated levels of extracellular signal-regulated kinase1/2 (ERK1/2), an important intracellular signal transduction molecule for neuronal function; cAMP response element-binding protein (CREB), a transcription factor that regulates neuronal function; and Akt, a critical factor in cell survival and apoptosis, in the hippocampus and cerebral cortex [11]. Moreover, the chronic administration of corticosterone at a dose of 32 mg/kg/day for 21 days [12] or the implantation of a corticosterone pellet (100 mg/kg/day for 21 days) [13] led to decreases in BDNF mRNA and protein levels in the hippocampus of the brain. These findings suggest that corticosterone-injected mice are a useful and reliable animal model for investigating the resilient effects of HMF on BDNF levels.

We used fluoxetine (FLX), a selective serotonin reuptake inhibitor (SSRI), as a positive control of an anti-depressant drug in the present study. FLX was previously shown to increase not only serotonin concentrations in the synaptic cleft, but also BDNF concentrations [7,14,15].

## 2. Results

### 2.1. Effects of Corticosterone and HMF on Body Weight Changes

**Figure 2:**
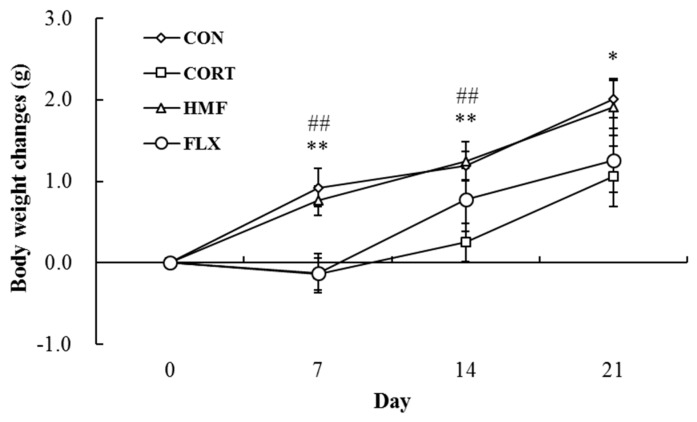
Body weight changes on Days 7, 14, and 21. Values are means ± SEM (*n* = 8). Symbols show significant differences between the following conditions: CON *vs.* CORT (* *p* < 0.05, ** *p* < 0.01) and CORT *vs.* CORT + HMF (^##^ *p* < 0.01).

Figure 2 shows changes in the body weights of mice during the experimental period, indicating that the repeated administration of corticosterone significantly induced decreases in body weight before Day 7, as reported previously [11,16]. Body weight gain by Day 21 in the CORT group (1.1 ± 0.4 g) was approximately 50% that in the CON group (2.0 ± 0.2 g). Figure 2 also shows that this decrease was attenuated by the administration of HMF; body weight gain in the CORT + HMF group was 1.9 ± 0.3 g on Day 21, which was similar to that in the CON group. This result indicated that HMF significantly suppressed corticosterone-induced body weight loss. Body weight gain in the CORT + FLX group on Day 21 was 1.3 ± 0.4 g, indicating that the representative anti-depressant drug, FLX, did not suppress body weight loss during the experimental period.

### 2.2. Effects of Corticosterone and HMF on Depressive-Like Behavior

**Figure 3:**
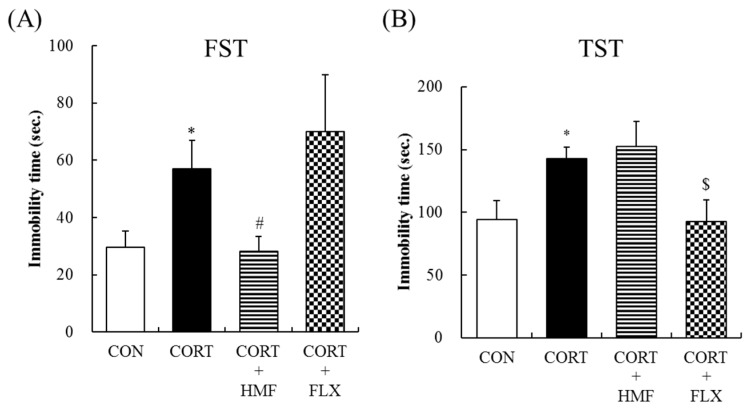
Effects of HMF on corticosterone-induced behavioral abnormalities in the forced swim test (**A**) and tail suspension test (**B**). Values are means ± SEM (*n* = 7–8). Symbols show significant differences between the following conditions: CON *vs.* CORT (* *p* < 0.05), CORT *vs.* CORT + HMF (^#^ *p* < 0.05), and CORT *vs.* CORT + FLX (^$^ *p* < 0.05).

The depressive-like behavior of mice was evaluated in the forced swim and tail suspension tests. Immobility times in the forced swim test were examined on Day 22. Figure 3A shows that the immobility time in the CORT group (57.1 ± 9.8 s) was approximately two-fold that in the CON group (29.6 ± 5.7 s), and also that the corticosterone treatment significantly (* *p* < 0.05) prolonged immobility times in the forced swim test. The immobility time in the CORT + HMF group was 28.1 ± 5.3 s, indicating that HMF significantly (^#^ *p* < 0.05) suppressed corticosterone-induced depression-like behavior. In this test, the immobility time in the CORT + FLX group (70.1 ± 19.8 s) was markedly longer than that in the CORT group, which is consistent with previous findings [17].

As another method to assess depressive-like behavior, immobility times in the tail suspension test were examined on Day 23. Figure 3B shows that the immobility time in the CORT group (142.6 ± 9.6 s) was significantly (* *p* < 0.05) longer than that in the CON group (94.5 ± 14.7 s). Figure 3B also shows that the administration of FLX significantly (^$^ *p* < 0.05) attenuated corticosterone-induced increases in immobility times, whereas HMF did not.

### 2.3. Effects of Corticosterone and HMF on the Expression of BDNF in the Hippocampus

**Figure 4:**
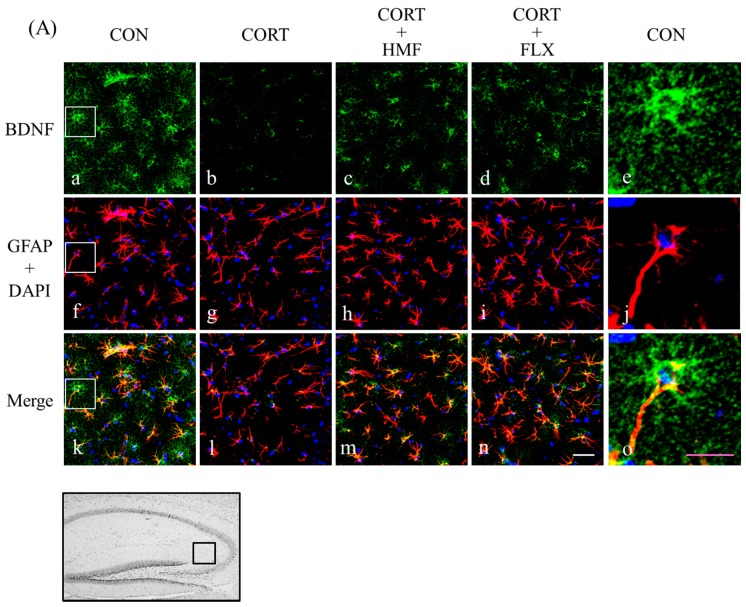

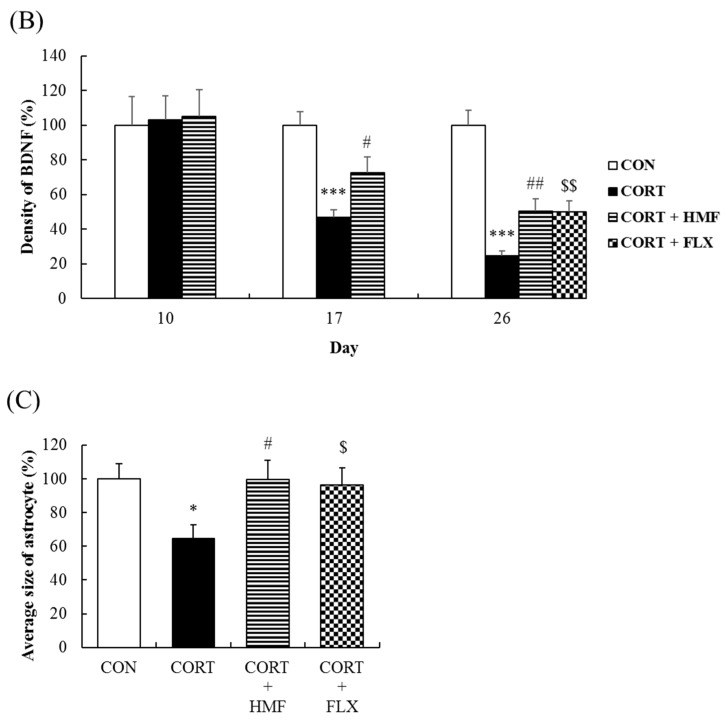
Effects of HMF on the expression of BDNF and GFAP immunoreactivity in the corticosterone-induced depressive mouse hippocampus. (A) Sagittal sections on Day 26 after continuous corticosterone injections were stained with specific antibodies, either anti-BDNF (green; a, b, c, d, e) or anti-GFAP with DAPI staining (red and blue, respectively; f, g, h, i, j). Each signal was merged in k, l, m, n and o, respectively. White squares in the CON group showed a typical astrocyte expressing BDNF, and each high-power magnification picture was shown as e, j, and o. The white and the pink scale bar show 50 µm and 25 µm, respectively. The location of the captured images in the hippocampus and quantification is shown with a square (0.22 mm^2^). (**B**) A quantitative analysis of BDNF-positive signal densities using ImageJ software. Values are means ± SEM (Day 10; *n* = 4, Day 17; *n* = 8, Day 26; *n* = 8–10). Symbols show significant differences between the following conditions: CON *vs.* CORT (*** *p* < 0.001), CORT *vs.* CORT + HMF (^#^ *p* < 0.05, ^##^ *p* < 0.01), and CORT *vs.* CORT + FLX (^$$^ *p* < 0.01). A quantitative analysis of the average size (**C**) of GFAP-positive signals on Day 26 using ImageJ software. Values are means ± SEM (*n* = 8–10). Symbols show significant differences between the following conditions: CON *vs.* CORT (* *p* < 0.05), CORT *vs.* CORT + HMF (^#^ *p* < 0.05), and CORT *vs.* CORT + FLX (^$^ *p* < 0.05).

Previous studies indicated that modifications in the expression of BDNF in the hippocampus may correlate with depression [7,18], and our previous findings demonstrated that HMF enhanced the synthesis of BDNF in the hippocampus of the ischemic brain [8,9]. Therefore, we investigated the effects of HMF on the expression of BDNF in the hippocampus on Days 10, 17, and 26 using an immunofluorescence method. Figure 4A shows representative photographs of the hippocampal region on Day 26. The BDNF signal on Day 26 was markedly weaker in the CORT group (b) than in the CON group (a), while those in the CORT + HMF group (c) and CORT + FLX group (d) were stronger than that in the CORT group. A quantitative analysis of BDNF-positive signal density (Figure 4B) showed that although BDNF signals in the CORT group were not weaker on Day 10, their densities gradually became weaker on Day 17; corticosterone significantly (*** *p* < 0.001) reduced the expression of BDNF at Day 26, while HMF and FLX significantly (^##^ *p* < 0.01 and ^$$^ *p* < 0.01, respectively) attenuated this decrease. These results indicated that HMF attenuated the corticosterone-induced suppression of BDNF expression in the hippocampus, similar to FLX.

Figure 4A also shows that BDNF-positive cells (a, b, c, d) and glial fibrillary acidic protein (GFAP; a marker of activated astrocytes)-positive cells with DAPI staining (f, g, h, i) had nearly merged (k, l, m, n), and also that the size of GFAP-positive cells was markedly smaller in the CORT group (g) than in the CON group (f), CORT + HMF group (h), and CORP + FLX group (i). Figure 4C shows that corticosterone significantly (* *p* < 0.05) reduced the size of GFAP-positive cells and that HMF and FLX significantly (^#^ *p* < 0.05 and ^$^ *p* < 0.05, respectively) attenuated this decrease. These results indicated that corticosterone inactivated astrocytes, resulting in a decrease in the expression of BDNF.

### 2.4. Effects of Corticosterone and HMF on Neurogenesis in the Hippocampus

**Figure 5:**
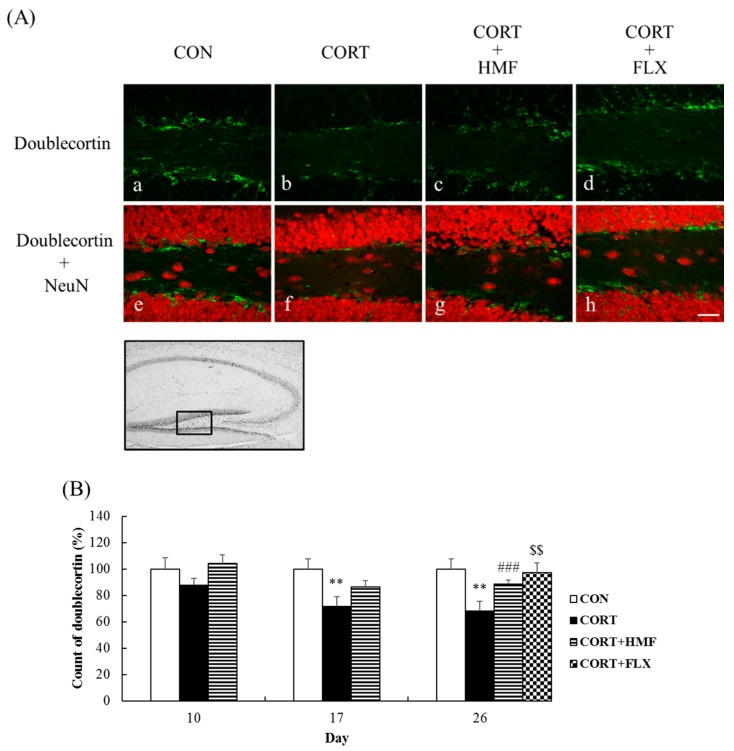
Effects of HMF on the expression of doublecortin immunoreactivity in the corticosterone-induced depressive mouse hippocampal dentate gyrus. (**A**) Sagittal sections on Day 26 after continuous corticosterone injections were stained with specific antibodies, either anti-doublecortin (green; a, b, c, d) or anti-NeuN (red; e, f, g, h). The scale bar shows 50 µm. The location of the captured images in the hippocampus is shown with a square (0.09 mm^2^). (**B**) A quantitative analysis of doublecortin-positive cell counts was performed manually. Values are means ± SEM (Day 10; *n* = 4, Day 17; *n* = 8, Day 26; *n* = 8–10). Symbols show significant differences between the following conditions: CON *vs.* CORT (** *p* < 0.01), CORT *vs.* CORT + HMF (^###^ *p* < 0.001), and CORT *vs.* CORT + FLX (^$$^ *p* < 0.01).

Decreases in hippocampal neurogenesis have been reported in corticosterone-treated rats [19] and chronic stress-loaded rats [20]. Therefore, we herein investigated the effects of HMF on neurogenesis in the subgranular zone of the dentate gyrus in the hippocampus using an anti-doublecortin (DCX) antibody, which recognizes a microtubule-associated protein expressed by neuronal precursor cells, on Days 10, 17, and 26. We defined and manually counted DCX-positive cells, which had more than 10μm diameter, in the hippocampal dentate gyrus. Figure 5A shows representative photographs of the hippocampal dentate gyrus region on Day 26, indicating that the DCX signal was markedly weaker in the CORT group (b) than in the CON group (a), CORT + HMF group (c), and CORT + FLX group (d). Figure 5B shows that the DCX signal was not significantly different among the three groups on Day 10, but was significantly (** *p* < 0.01) weaker in the CORT group on Day 17. It was also significantly (** *p* < 0.01) weaker than that in the CON group on Day 26, while the HMF and FLX treatments significantly attenuated these changes (^###^ *p* < 0.001 and ^$$^ *p* < 0.01, respectively). These results indicated that HMF and FLX attenuated corticosterone-induced reductions in neurogenesis.

### 2.5. Effects of Corticosterone and HMF on the Neuronal Network in the Hippocampus

**Figure 6:**
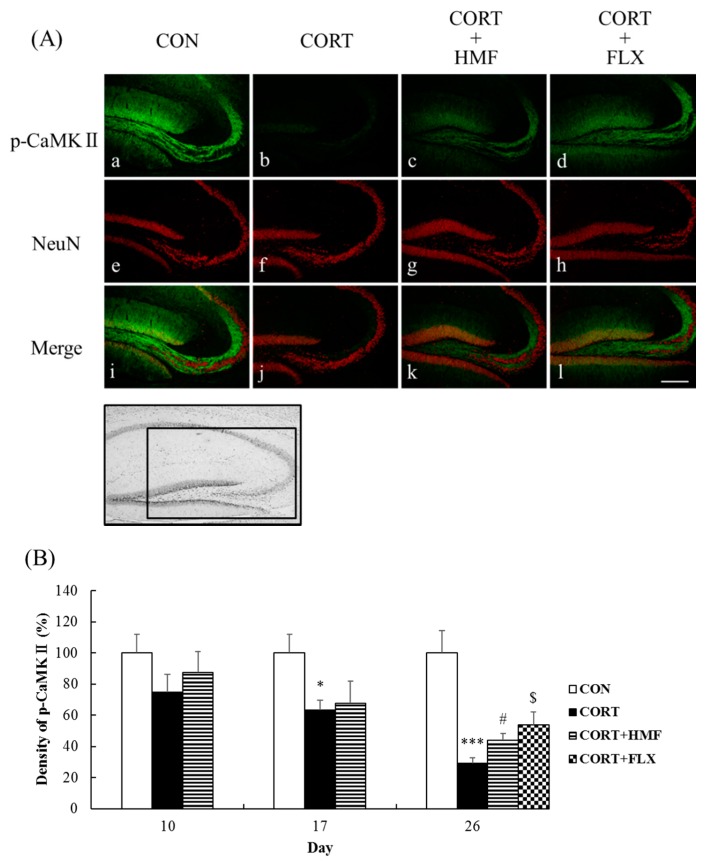
Effects of HMF on the expression of phosphorylated CaMK II and NeuN immunoreactivity in the corticosterone-induced depressive mouse hippocampus. (**A**) Sagittal sections on Day 26 after continuous corticosterone injections were stained with specific antibodies, either anti-phospho-CaMK II (green; a, b, c, d) or anti-NeuN (red; e, f, g, h). Each signal was merged in i, j, k and l, respectively. The scale bar shows 200 µm. The location of the captured images in the hippocampus and quantification is shown with a square (1.0 mm^2^). (**B**) A quantitative analysis of phospho-CaMK II-positive signal density using ImageJ software. Values are means ± SEM (Day 10; *n* = 4, Day 17; *n* = 8, Day 26; *n* = 8–10). Symbols show significant differences between the following conditions: CON *vs.* CORT (* *p* < 0.05, *** *p* < 0.001), CORT *vs.* CORT + HMF (^#^ *p* < 0.05), and CORT *vs.* CORT + FLX (^$^ *p* < 0.05).

We evaluated the effects of corticosterone and HMF on the neuronal network in the hippocampus using experiments with an anti-phosphorylated calcium-calmodulin-dependent protein kinase II (p-CaMK II) antibody. CaMK II is one of the serine/threonine protein kinases, the autophosphorylation of which is known to be important for neuroplasticity [21]. Figure 6A shows representative photographs of the hippocampus on Day 26, indicating that the p-CaMK II signal was markedly weaker in the CORT group (b) than in the CON group (a). Figure 6A also shows that corticosterone-induced reductions in the intensity of the p-CaMK II signal were attenuated in the CORT + HMF group (c) and CORT + FLX group (d). The signal of NeuN, a neuronal marker, was similar among the four groups (e, f, g, h) on Day 26, suggesting that corticosterone did not injure neuronal cells.

Figure 6B shows that the p-CaMK II signal was not significantly different among the three groups on Day 10, but was significantly (* *p* < 0.05) weaker in the CORT group on Day 17 (63.2% ± 6.2% of CON group). On Day 26, the p-CaMK II signal was significantly (*** *p* < 0.001) weaker in the CORT group than in the CON group, and this change was attenuated by the HMF and FLX treatments (^#^ *p* < 0.05 and ^$^ *p* < 0.05, respectively).

### 2.6. Effects of Corticosterone and HMF on ERK1/2-Phosphorylation in the Hippocampus

**Figure 7:**
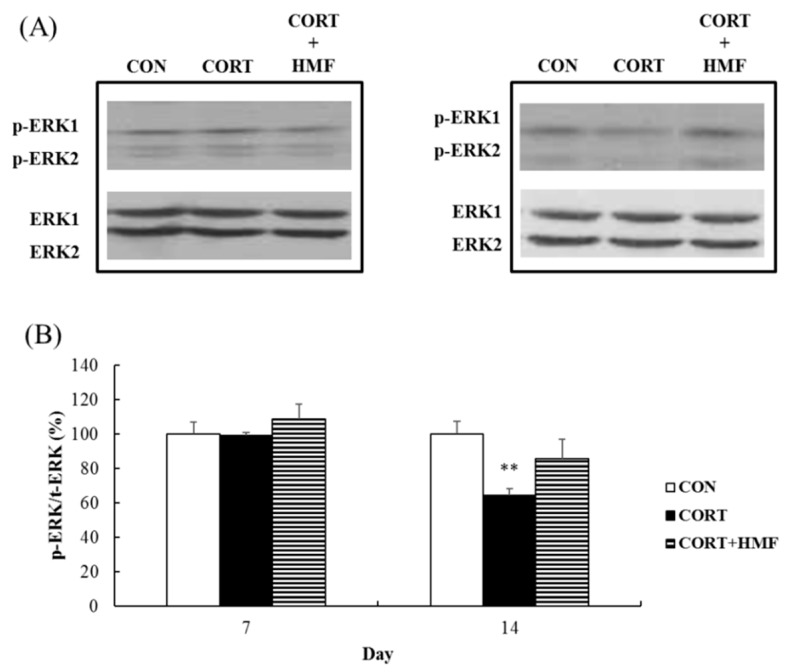
A Western blot analysis of the influence of HMF on the expression of phosphorylated ERK in the corticosterone-induced depressive mouse hippocampus. (**A**) Representative band patterns of p-ERK1/2 and ERK1/2. (**B**) A quantitative analysis of the p-ERK/ERK ratio using ImageJ software. Values are means ± SEM (Day 10; *n* = 4, Day 17; *n* = 5). Symbols show significant differences between the following conditions: CON *vs.* CORT (** *p* < 0.01).

ERK1/2 has been implicated in the depression-like symptoms elicited by stress-related insults [22,23]. We previously reported that HMF activates (phosphorylates) ERK1/2 in cortical neurons *in vitro* [24] and in the hippocampus *in vivo* [9], while corticosterone decreases the level of phosphorylated ERK1/2 (p-ERK1/2) [11]. Therefore, we herein investigated the effects of HMF on the activation of ERK1/2 in corticosterone-treated brains using a western blot analysis. The band intensity of p-ERK1/2 was not influenced by corticosterone or HMF on Day 10 (Figure 7A), whereas that in the CORT group significantly (** *p* < 0.01) reduced on Day 17 and the HMF treatment slightly restored phosphorylation levels (Figure 7B). These results suggested that HMF attenuated corticosterone-induced decreases in the activation of ERK1/2.

## 3. Discussion

Several theories exist for the basis of depression. The monoamine hypothesis of depression suggests that mood disorders including depression are caused by an imbalance in monoamines, particularly serotonin, in the brain, and this imbalance may be corrected by the administration of anti-depressant drugs [25]. On the other hand, the neurotrophin hypothesis of depression suggests that a decrease in neurotrophins, particularly BDNF, is an important cause of depression, and that anti-depressant drugs exert their effects by increasing BDNF levels [7]. The neuroplastic hypothesis of depression suggests that alterations in the plasticity of neural networks are a relevant factor in mood disorders including depression [26], and FLX restores structural plasticity [27]. The cytokine hypothesis of depression implicates the immune system in the development of depression, and suggests that anti-depressant drugs prevent microembolism-induced changes in inflammation and behavior [28,29].

By focusing on the neurotrophin (BDNF) hypothesis of depression, we herein demonstrated that the HMF treatment attenuated corticosterone-induced reductions in BDNF levels in the hippocampus (Figure 4) as well as corticosterone-induced depression-like symptoms (Figure 2 and Figure 3), suggesting that HMF has potential as an anti-depressant agent. BDNF is the most abundant neurotrophic factor in the brain and plays an important role not only in neural development, survival, and function, but also in neurogenesis and neuroplasticity, both of which are important targets for depressive disorder treatments [30]. Neurogenesis in the hippocampus was previously reported to be suppressed during depression [19,20], and chronic treatments with SSRIs increased the expression of BDNF, proliferation/differentiation of neuronal progenitor cells, and maturation of newborn neurons [31]. We showed that the HMF treatment attenuated corticosterone-induced reductions in the expression of DCX in the hippocampus (Figure 5). Neuroplasticity in the hippocampus is also known to be suppressed during depression [32,33], and anti-depressant drugs induce plastic changes in neuronal connectivity, which gradually lead to improvements in neuronal information processing and mood recovery. In the present study, we demonstrated that the HMF treatment attenuated corticosterone-induced reductions in the expression of p-CaMK II in the hippocampus (Figure 6).

Consistent with previous findings showing that SSRIs activate glial cells, particularly astrocytes [34], we found that the HMF treatment attenuated the corticosterone-induced inactivation of astrocytes in the hippocampus (Figure 4A and C). The results of the immunohistochemical study (Figure 4A) revealed that BDNF-positive cells merged with GFAP-positive cells. Collectively, our results and previous findings showed that HMF and SSRIs enhance neurogenesis and neuroplasticity in the depressive hippocampus via BDNF synthesized by astrocytes, and we are now investigating the mechanisms responsible for the effects of HMF on astrocytes *in vitro*.

Commonly used depressive-like behavioral tests are the forced swim test and tail suspension test, with increases in immobility times reflecting a depressive state. The results of the present study showed that the HMF treatment decreased corticosterone-induced increases in immobility times in the forced swim test, but not in the tail suspension test. In contrast, the FLX treatment decreased immobility times in the tail suspension test, but not in the forced swim test (Figure 3). This inconsistency suggests that HMF attenuates depressive-like behavior through different mechanism(s) to those of FLX.

A previous study that examined postmortem tissues demonstrated that hippocampal volumes were significantly smaller in patients with major depressive disorders [35]. The findings of a neuroimaging study also revealed smaller hippocampal volumes in depressed patients that were attenuated by an anti-depressant treatment [36]. We herein found that the average size of GFAP-positive cells were significantly reduced in the hippocampus in the CORT group, but were attenuated by the HMF treatment (Figure 4). Since 1) the total number, somal volume, and protrusion length of GFAP-positive astrocytes correlate with hippocampal volume [37], and 2) astrocytes contribute to enhancements in neurotrophic support and associated augmentations in synaptic plasticity [38], the HMF treatment may effectively maintain astrocyte function, and this may be followed by the production of BDNF.

On the other hand, ERK1/2, a signal transduction factor for several receptors, was previously reported to contribute to depression. The treatment of mice with corticosterone was previously shown to selectively decrease p-ERK1/2 levels in the dentate gyrus, but not in the CA1/CA3 regions; therefore, decreases in the levels of p-ERK1/2 temporally coincided with depressive-like behavioral responses [22]. The phosphorylation level of ERK1/2 may mediate the efficacy of anti-depressant drugs in depressed humans and animal models of depression [23]. We herein showed that the HMF treatment attenuated corticosterone-induced reductions in p-ERK1/2 levels in the hippocampus (Figure 7).

The results of the present study suggest the potential of HMF as a novel anti-depressant drug based on the “BDNF hypothesis of depression”.

## 4. Materials and Methods

### 4.1. Animals

Male C57BL/6 strain mice (seven weeks old) were purchased from Japan SLC, Inc. (Hamamatsu, Shizuoka, Japan). Stock diets and tap water were freely available during the experimental period. Mice were kept at 23 ± 1 °C on a 12-h light/dark cycle (lights on 8:00–20:00). All animal experiments were carried out in accordance with the Guidelines for Animal Experimentation and approved by the Animal Care and Use Committee of Matsuyama University. Mice were divided into 10 experimental groups, and each group contained 5–8 mice.

### 4.2. Administration of Corticosterone and Test Drugs

Mice in the seven groups were subcutaneously (s.c.) administered corticosterone (Wako Pure Chemical Industries, Ltd., Osaka, Japan) at a dose of 20 mg/kg/day in a volume of 5 mL/kg once a day. The administration periods used were 9 days for two groups, 16 days for another two groups, and 25 days for the last three groups. The remaining three groups were administered vehicle (DMSO/polyethylene glycol (PEG)-300 (3:7) solution). The administration periods used were 9 days, 16 days, and 25 days for each group.

HMF was prepared from orange oil (Wako Pure Chemical Industries, Ltd., Osaka, Japan) as described previously [24]. FLX was purchased from LKT Laboratories, Inc. (St. Paul, MN, USA). Both chemicals were dissolved in DMSO/PEG-300 (3:7) solution. In the corticosterone plus HMF-treated group (CORT + HMF group) or corticosterone plus FLX-treated group (CORT + FLX group), HMF (50 mg/kg/day) or FLX (10 mg/kg/day) was s.c. administered simultaneously with corticosterone for 9, 16, or 25 days. The control group (CON group) and corticosterone-treated group (CORT group) were s.c. administered the same volume of vehicle (DMSO/PEG-300 solution).

### 4.3. Forced Swim Test

On Day 22, 30 min after the administration of corticosterone/test drugs, mice were subjected to the forced swim test with a minor modification to the methods described by Porsolt [39]. Mice were individually placed in the center of a plastic cylinder (10 (Φ) × 25 (H) cm) filled with water (up to 15 cm in height) at an ambient temperature, and allowed to swim freely for 6 min. Immobility times were manually counted during the last 4 min. Mice were judged to be immobile when only making small movements necessary, namely, moving their limbs subtly, in order to keep their heads above the water and also when floating.

### 4.4. Tail Suspension Test

On Day 23, mice were subjected to the tail suspension test according to previously described methods [40] 30 min after the administration of corticosterone/test drugs. Mice were individually suspended 10 cm above a tabletop with a peg positioned 2 cm from the tip of the tail for 6 min, and immobility times were manually counted in the last 4 min. Mice were judged to be immobile when they hung passively and completely motionless.

### 4.5. Immunofluorescence for Confocal Microscopy

Mice were transcardially perfused with ice-cold phosphate buffered-saline (PBS) 1 day after the final administration of samples (Day 10, 17, or 26), and their brains were used in immunohistochemical and biochemical analyses. In the immunohistochemical analysis, brains were postfixed as previously described [41], and sagittal sections at 30 μm were incubated with the following primary antibodies; mouse anti-GFAP (1:200; Sigma-Aldrich, St. Louis, MO, USA), rabbit anti-BDNF (dilution 1:150; Epitomics, Burlingame, CA, USA), mouse anti-NeuN (1:300; Millipore, Billerica, MA, USA), rabbit anti-phospho CaMK II (p-Thr286, 1:500; Sigma-Aldrich), and goat anti-DCX (1:50; Santa Cruz Biotechnology, Santa Cruz, CA, USA). As secondary antibodies, Alexa Fluor 488 goat anti-rabbit IgG (H + L) (1:300; Invitrogen, Carlsbad, CA, USA), Alexa Fluor 488 donkey anti-goat IgG (H + L) (1:300), Alexa Fluor 568 goat anti-rabbit IgG (H + L) (1:300), and Alexa Fluor 568 goat anti-mouse IgG (H + L) (1:300) were used. A mounting medium with DAPI was employed (Vectashield; Vector Laboratories, Burlingame, CA, USA), and images were captured with a confocal fluorescence microscopy system (LSM510; Zeiss, Oberkochen, Germany). ImageJ software (NIH, Bethesda, Rockville, MD, USA) was used for the quantification of fluorescence signals for BDNF and phospho-CaMK II in images as described previously [8]. The fluorescence signals of doublecortin-positive cells were manually counted with a confocal fluorescence microscopy system. The location of the captured images and quantification is shown with a square in each figure.

### 4.6. Western Blot Analysis

Hippocampal regions after perfusion were weighed and homogenized in 10 volumes of RIPA buffer (20 mM Tris-HCl, pH 7.5, 0.1% SDS, 150 mM NaCl, 1% NP-40, 1% sodium deoxycholate, 2 mM EDTA, and a protease inhibitor cocktail (Roche, Mannheim, Germany)). Lysates were then centrifuged at 20,000× *g* at 4 °C for 30 min, and supernatant solutions were collected as the protein extract. SDS-polyacrylamide gel electrophoresis was used to separate equal amounts of protein (20 µg), which were then electroblotted onto an Immuno-Blot^TM^ PVDF Membrane (Bio-Rad, Hercules, CA, USA) as previously described [9]. The primary antibodies used were rabbit antibodies against 44/42 ERK1/2 (Millipore), which recognize 44-kDa ERK1 and 42-kDa ERK2, and phospho-44/42 MAPK (Thr202/Tyr204; Cell Signaling, Woburn, MA, USA), which recognize phosphorylated ERK1 and ERK2. The secondary antibody was horseradish peroxidase-linked anti-rabbit IgG (Cell Signaling). Immunoreactive bands were visualized by ECL-prime (GE Healthcare, Chalfont St. Giles, UK), and band intensities were measured using a LAS-3000 imaging system (Fujifilm, Tokyo, Japan).

### 4.7. Statistical Analysis

Data for individual groups were expressed as means ± SEM. Data were analyzed using an unpaired *t*-test, and a value of *p* < 0.05 was considered significant.

## 5. Conclusions

HMF attenuated corticosterone-induced body weight loss, corticosterone-induced depressive-like behavior, the corticosterone-induced down-regulation of BDNF and GFAP, and corticosterone-induced reductions in the expression of DCX, p-CaMK II, and ERK1/2 in the hippocampus. These results suggest that HMF enhances the production of BDNF in the hippocampus, resulting in the attenuation of corticosterone-induced depression through enhancements in the production of BDNF.
